# Physical and functional interaction of Rnf2 with Af9 regulates basal and aldosterone-stimulated transcription of the *α-ENaC* gene in a renal collecting duct cell line

**DOI:** 10.1042/BSR20130086

**Published:** 2013-10-25

**Authors:** Zhi-Yuan Yu, Qun Kong, Bruce C. Kone

**Affiliations:** *Department of Internal Medicine, The University of Texas Medical School at Houston, Houston, TX 77030, U.S.A.

**Keywords:** aldosterone, epigenetics, H2AK119, H3K27, methyltransferase, Polycomb, Af9, ALL1-fused gene from chromosome 9 protein, AFF1, AF4/FMR2 family member 1, CBX8, chromobox protein 8, CD, collecting duct, ChIP, chromatin immunoprecipitation, CNT, connecting tubule, DAPI, 4′,6-diamidino-2-phenylindole, Dot1a, disruptor of telomeric silencing isoform 1a, ECL, enhanced chemiluminescence, EGFP, enhanced green fluorescent protein, ENaC, epithelial sodium channel, EZH2, enhancer of zeste homologue 2, FBS, fetal bovine serum, GAPDH, glyceraldehyde-3-phosphate dehydrogenase, GRE, glucocorticoid responsive element, GST, glutathione transferase, HRP, horseradish peroxidase, H2AK119ub1, histone H2A lysine 119 monoubiquitination, H3K27me3, trimethylated histone H3 lysine 27, H3K79me3, histone H3 lysine 79 trimethylated, MR, mineralocorticoid receptor, PcG, Polycomb group, Per1, Period 1, PH, polyhomeotic, PRC, Polycomb repressive complex, QPCR, quantitative PCR, qRT-PCR, quantitative real-time PCR, Rnf, RING finger protein, SEC, super elongation complex, siRNA, small interfering RNA

## Abstract

The physical and functional interaction of Rnf2 (RING finger protein 2), a central component of the PRC (Polycomb repressive complex) 1 and Af9 (ALL1-fused gene from chromosome 9 protein), an aldosterone-sensitive transcription factor, in regulating basal and aldosterone-stimulated transcription of the *α-ENaC* (epithelial Na^+^ channel α-subunit) gene was explored in mIMCD3 CD (collecting duct) cells. Since Rnf2 lacks DNA-specific binding activity, other factors must mediate its site-specific chromatin recruitment. Rnf2 and Af9 co-localized in the nucleus and co-immunoprecipitated. A GST (glutathione transferase)–Af9 carboxy-terminal fusion protein directly interacted with *in vitro* translated Rnf2 in GST pull-down assays. Rnf2 knock down enhanced basal and aldosterone-stimulated α-ENaC mRNA levels and α-ENaC promoter activity. ChIP/QPCR (chromatin immunoprecipitation/quantitative PCR) assays demonstrated enrichment of Rnf2, H2AK119 (mono-ubiquitinated histone H2A lysine 119), and H3K27me3 (histone H3 lysine 27 trimethylated), a PRC2 chromatin mark, at multiple α-ENaC promoter subregions corresponding to regions of known Af9 enrichment, under basal conditions. Sequential ChIP confirmed Rnf2–Af9 co-occupancy of the α-ENaC promoter. Aldosterone provoked early and sustained depletion of Rnf2, ubiquitinated H2AK119, and trimethylated H3K27 associated with the subregions of the α-ENaC promoter. Thus, Af9 mediates site-selective physical and functional recruitment of Rnf2 to the α-ENaC promoter to constrain basal α-ENaC transcription in collecting duct cells, and aldosterone reverses this process.

## INTRODUCTION

The ENaC (epithelial Na^+^ channel) expressed in the apical membrane of the renal CD (collecting duct) mediates the rate-limiting step in active Na^+^ and fluid absorption in this segment and thereby contributes to the control of extracellular fluid volume and BP (blood pressure) [1]. ENaC consists of α-, β- and γ-subunits, which are encoded by the unlinked *Scnn1a* (‘α-ENaC’ in this report), *Scnn1b* and *Scnn1c* genes. Of these subunit genes, *α-ENaC* appears to be critical to overall salt balance, as evidenced by the severe renal salt wasting exhibited by mice with targeted inactivation of α-ENaC in the CNT (connecting tubule)/CD [2]. α-ENaC is also a molecular target for aldosterone. Aldosterone induction of ENaC functional activity is strictly dependent on the level of α-ENaC expression in the CD principal cells. Indeed, aldosterone administration or secondary hyperaldosteronism resulting from a low-Na^+^ diet increases *α-ENaC* gene transcription without increasing β- or γ-subunit expression or α-ENaC mRNA stability [3].

*α-ENaC* gene transcription is under complex genomic and epigenomic controls that have yet to be fully characterized. A working model posits that under basal conditions, *α-ENaC* gene transcription is active, but constrained by combinatorial interactions of the H3K79 (histone H3 lysine 79) methyltransferase Dot1a (disruptor of telomeric silencing isoform 1a) with Sirt1 [4] or Af9 (ALL1-fused gene from chromosome 9 protein) [5–7]. As evidence of the importance of these epigenetic controls on α-ENaC regulation, mice with CNT/CD-specific targeted inactivation of Dot1a were found to exhibit much greater α-ENaC mRNA levels compared with controls [8]. Af9 associates with each of four partially overlapping subregions of the α-ENaC promoter designated R0 (−988/−713), R1 (−735/−415), R2 (−414/+80), and R3 (−57/+439) [6]. Thus far, an Af9 *cis*-element has been characterized at +78/+92 in the R3 subregion of the α-ENaC promoter, and it contributes, through its interaction with, and the actions of, Dot1a, to basal repression of α-ENaC transcription in the immortalized inner medullary CD cell line, mIMCD3 [8]. The potential clinical relevance of this epigenetic pathway to human salt balance was highlighted by a recent exploratory analysis that suggested an association between a human AF9 SNP (single nucleotide polymorphism) and untreated hypertension in African-Americans [9].

Aldosterone induction of *α-ENaC* gene transcription also involves genomic and epigenomic mechanisms. It has long been known that aldosterone, liganded to the MR (mineralocorticoid receptor), binds to a GRE (glucocorticoid responsive element) at −811 of the murine *α-ENaC* gene to *trans*-activate it [10]. Aldosterone, also apparently through interaction with nuclear hormone receptors, triggers binding of the circadian clock protein Per1 (Period 1) to at least one of four E-box elements to *trans*-activate the α-ENaC promoter [11–13]. However, nuclear hormone receptor-independent effects were revealed when mice with CNT/CD-specific knockout of the MR did not develop the severe salt-wasting phenotype [14] observed with α-ENaC knockout in these same segments [2]. We discovered that aldosterone also down-regulates and disperses the Dot1a–Af9 complex from the α-ENaC promoter, resulting in de-repression of α-ENaC transcription [5–7]. Whether other transcriptional repressors contribute to the epigenetic repression and aldosterone-induced de-repression of *α-ENaC* gene transcription is unknown.

Af9 is known to partner with other proteins, including CBX8 (chromobox protein 8) [15], a member of the PcG (Polycomb group) proteins that serve as epigenetic repressors in embryonic development, cellular differentiation, stem cell self-renewal and neoplasia [16,17]. CBX8 complexes with Rnf2 (RING finger protein 2), together with Bmi1 and a variable number of associated proteins such as Rnf1, PH (polyhomeotic)1 and PH2, to form PRC (Polycomb repressive complex)1. Rnf2 functions as an E3 ubiquitin ligase within PRC1, and silences genes through H2AK119ub1 (histone 2A K119 monoubiquitination) [18], nucleosome compaction [19] and/or suppression of local H3K4me3 (histone H3 lysine 4 trimethylated) deposition and loading of RNA polymerase II [20]. Another complex, PRC2 [16], contains the conserved histone methyltransferase EZH2 (enhancer of zeste homologue 2), which catalyses H3K27 di- and trimethylation [21]. A specific, direct interaction of Af9 with Rn2 has not been described, nor has the potential impact of PRC1 or PRC2 components on α-ENaC transcription been addressed.

The present study was designed to test the hypothesis that Rnf2, through direct or indirect interactions with Af9, contributes to the basal repression and aldosterone-induced de-repression of α-ENaC transcription in mIMCD3 cells. The results indicate that, in contrast with the prior studies in yeast, Rnf2 directly interacts with Af9 in mIMCD3 cells under basal conditions and associates with multiple subregion of the α-ENaC promoter along with the PRC1 and PRC2 signatures H2AK119ub1 and H3K27me3 (histone H3 lysine 27 trimethylated). Aldosterone triggers loss of Rnf2 and these histone marks from the R1–R3 subregions of the α-ENaC promoter. These results expand the complexity of the epigenetic repression/de-repression model of α-ENaC transcription and reveal a novel physical and functional interaction of Af9 and Rnf2 that likely is more broadly applicable to the regulation of many other genes.

## MATERIALS AND METHODS

### Reagents and plasmids

Aldosterone was from Sigma. The SYBR® GreenER qPCR SuperMix Universal and Lipofectamine™ 2000 reagent were purchased from Invitrogen. Several plasmids have been described [5–7]: pGL3-basic-1.3α-ENaC contains the murine α-ENaC promoter fused to the gene encoding firefly luciferase; pcDNA3.1-Zeo-1.3α-ENaC-Luc contains the 1.3 kb α-ENaC promoter and luciferase-coding cassette from pGL3-basic-1.3α-ENaC subcloned into pcDNA3.1-Zeo, GST (glutathione transferase)–Af9(397-557) is a GST fusion protein encoding the C-terminal amino acids 397–557 of Af9, and has been shown to interact with Dot1a [6]. The encoding DNA for mouse Rnf2 (accession MGC 28374) was PCR-amplified using forward primer 5′-ATGTCTCAGGCTGTGCAGAC-3′ (nts 81–100) and reverse primer 5′-TCATTTGTGCTCCTTGGTGGG-3′ (nts 1071-1091) and subcloned into the *Xho*I/*Hind*III sites of pcDNA3.1-Zeocin to create pcDNA3.1-Zeo-Rnf2 and into the *ECoR*I/*Xho*I sites of pEGFP-C3 to create pEGFP-Rnf2. The Af9 encoding DNA was subcloned into pDsRed to create pDsRed-Af9. DNA sequencing and restriction mapping verified the authenticity of these constructs. Antibodies directed against Rnf2 (Abcam), H2AK119ub1 (Millipore), and H3K27me3 (Abcam) were purchased from commercial vendors. Rnf2 siRNAs (small interfering RNAs) and control siRNA were from Sigma.

### mIMCD3 cell culture, aldosterone treatment, transient and stable transfections, and luciferase assays

mIMCD3 cell culture, aldosterone treatment, transient and stable transfections and luciferase assays of promoter activity were performed as described [6,7]. In brief, mIMCD3 cells were cultured at 37°C in a 5% CO_2_ environment in DMEM (Dulbecco's modified Eagle's medium)/F-12 plus 10% FBS (fetal bovine serum). For experiments involving aldosterone, the cells were cultured in medium of the same composition, except containing 10% charcoal-stripped FBS for at least 24 h, before adding 1 μM aldosterone or 0.01% ethanol as vehicle control. Cell lines stably transfected with pcDNA3.1-Zeo-1. 3α-ENaC-Luc have been characterized [8]. Promoter activities in the stable cell lines were assayed using the Dual-Luciferase Reporter Assay System (Promega) and were normalized to cell protein content. siRNA knockdown was performed using the Lipofectamine 2000 reagent (Life Technologies) and control and Rnf2-specific siRNAs by methods previously described [8].

### Co-immunoprecipitation and immunoblotting

Co-immunoprecipitation and immunoblotting were performed as we previously described [6]. Briefly, whole cell lysates of mIMCD3 cells were prepared by using RIPA buffer (PBS containing 0.1% SDS, 0.5% sodium deoxycholate, 1% Nonidet P40, 1 mM Na_3_VO_4_ (sodium orthovanadate), 1 mM PMSF and 3% protease inhibitor cocktail). After preclearing with IgG plus protein A/G-Sepharose beads, the lysates were incubated with anti-Af9 antibody or isotype control IgG in RIPA buffer overnight at 4°C, followed by the addition of protein A/G-agarose beads for an addition 1 h with rotation at 4°C. The beads were washed four times with RIPA buffer, and the precipitates were eluted with Laemmli sample buffer, boiled for 5 min and analysed by 4–20% SDS/PAGE. Proteins were electrophoretically transferred to PVDF membranes (Hybond ECL (enhanced chemiluminescence), Amersham) and subjected to immunoblot analysis with anti-Rnf2 antibody. The blots were washed extensively with a solution containing 50 mM Tris, pH 8.0, 138 mM NaCl, 2.7 mM KCl, and 0.05% Tween 20. The antigen-antibody complexes were detected by the ECL protocol using HRP (horseradish peroxidase)-conjugated donkey anti-rabbit IgG as secondary antibody.

### GST pull-down assay

The GST–Af9-(397–557) fusion protein was purified from sonicates of IPTG (isopropyl β-d-thiogalactoside)-induced DH5α bacterial cells according to the manufacturer's instructions (Amersham Pharmacia Biotech) and incubated with 50 μl of glutathione-Sepharose 4B beads for 1 h at 4°C. After centrifugation, the pellets were collected and resuspended in lysis buffer (PBS containing protease inhibitor cocktail). For the *in vitro* binding reaction, 20 μl of purified, bead-bound GST or GST–Af9-(397–557) was incubated in protein-binding buffer (20 mM Tris, pH 8.0; 150 mM KCl; 1 mM EDTA; 4 mM MgCl_2_; 0.2% Nonidet P40; 10% glycerol) with 10 μl of methionine-unlabeled translation product (TNT Quick Coupled Transcription/Translation Systems, Promega) of full-length Rnf2 overnight at 4°C. The samples were then washed four times in binding buffer. The bound proteins were liberated by boiling in Laemmli sample buffer and were analysed by SDS/PAGE. The proteins were electrophoretically transferred to PVDF membranes (Hybond ECL, Amersham Biosciences). The blots were probed with an anti-Rnf2 antibody (0.2 μg/ml) overnight at 4°C. The blots were washed extensively with a solution containing 50 mm Tris, pH 8.0, 138 mm NaCl, 2.7 mm KCl, and 0.05% Tween 20. The antigen-antibody complexes were detected by the ECL protocol using HRP-conjugated donkey anti-rabbit IgG as secondary antibody.

### Immunofluorescence microscopy

mIMCD3 cells grown on glass coverslips were transiently co-transfected with pEGFP-Rnf2 and pDsRed-Af9. After 24 h, the cells were fixed in buffered 4% formaldehyde for 10 min. After 3 washes in Dulbecco's PBS, slides were stained with DAPI (4′,6-diamidino-2-phenylindole) mounted with Vectashield mounting medium (Vector Laboratories) and visualized with an inverted microscope equipped with phase and fluorescence optics (Nikon Eclipse Ti microscope, Nikon Instruments). Collected images were processed with Photoshop (Adobe Systems).

### ChIP/QPCR (chromatin immunoprecipitation/quantitative PCR), re-ChIP and RT-qPCR

ChIP/QPCR and re-ChIP/QPCR were performed and analysed essentially as previously described [5,7] using the ChiP-IT Express Enzymatic Kit and re-ChIP-IT Kit (Active Motif), except that antibodies directed against Rnf2, H2AK119ub1 and H3K27me3 were used. For ChIP assays, mIMCD3 cells were fixed with formaldehyde, harvested and chromatin was enzymatically sheared according to the manufacturer's protocol. Chromatin immune precipitates were isolated on protein G magnetic beads using 2 μg of Rnf2, H2AK119ub1 and H3K27me3 antibodies or 2 μg IgG. After washing, the ChIPs were eluted, reverse cross-linked, and subjected to agarose gel analysis and QPCR. For re-ChIP, chromatin immune precipitates from the first ChIP were desalted over columns, incubated with the protein G magnetic beads and second antibody or IgG, and processed as above. ChIPs were normalized to input DNA and the IgG control. RT-qPCR to measure α-ENaC, Rnf2 and GAPDH (glyceraldehyde-3-phosphate dehydrogenase) (as a housekeeping control) mRNA levels in miMCD3 cells utilized methods and primers as previously described [22].

### Data analysis

Quantitative data are expressed as means±S.E.M. and were analysed for statistical significance by one-way ANOVA or Student's *t*-test as appropriate. *P* values <0.05 were taken as significant.

## RESULTS

### Rnf2 interacts with Af9 *in vitro* and in mIMCD3 cells

**Figure 1:**
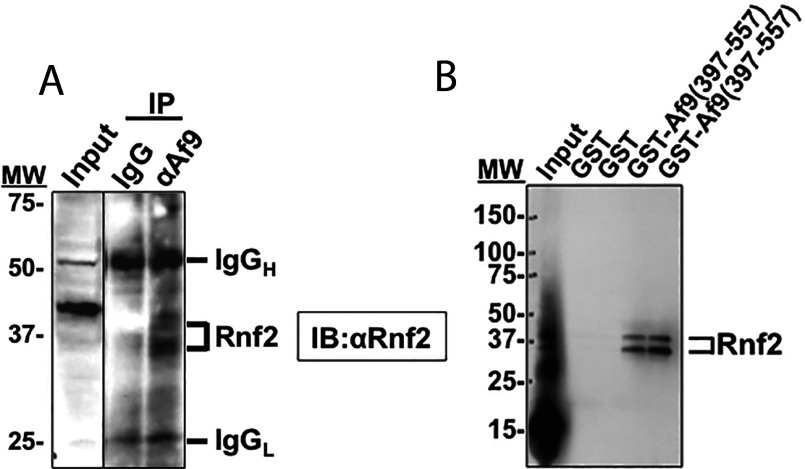
Rnf2 and Af9 interact *in vitro* and *in vivo* (**A**) Co-immunoprecipitation assay demonstrating that endogenous Rnf2 and Af9 proteins in mIMCD3 cells are present in the same protein complex. Whole cell lysates of mIMCD3 cells were IP (immunoprecipitated) with anti-Af9 antibody or IgG (as a negative control) as detailed in ‘Materials and Methods.’ IP proteins were further resolved on IBs (immunoblots) probed with anti-Rnf2 antibody. The IB is representative of three independent experiments. Positions of MW (molecular weight) markers are indicated. The Rnf2 doublet likely reflects unmodified and autoubiquitinated forms of endogenous Rnf2 [23]. (**B**) GST pull-down assay showing interaction of *in vitro* translated Rnf2 with amino acids 397–557 of Af9. GST and GST–Af9-(397–577) fusion protein were purified from *E. coli* and incubated with *in vitro* translated Rnf2. Proteins bound to glutathione-Sepharose 4B beads were examined by IB analysis with anti-Rnf2 antibody (n=3). Positions of MW markers are indicated. The Rnf2 doublet likely reflects unmodified and autoubiquitinated forms of Rnf2 [23] generated during *in vitro* translation with the reticulocyte lysate system [24].

To determine if endogenous Rnf2 interacts with Af9 in mIMCD3 cells, co-immunoprecipitation experiments with anti-Af9 antibody or isotype control IgG (as a negative control) were performed. The precipitated immune complex was further analysed by immunoblotting with anti-Rnf2 antibody to identify Rnf2 in the complex. As shown in Figure 1A, Rnf2 was immunoprecipitated by the anti-Af9 antibody but not with non-immune IgG. The Rnf2 doublet likely reflects unmodified and autoubiquitinated forms of endogenous Rnf2 [23]. Reciprocal co-immunoprecipitation with anti-Rnf2 antibody followed by immunoblotting with anti-Af9 antibody was performed, but since Af9 comigrates with IgG heavy chain at about 50 kDa, discrimination of an Af9 band was impossible. To verify the Rnf2–Af9 interaction by an independent method, and to determine whether the interaction was direct rather than mediated by a bridging protein in a ternary complex as suggested in yeast two-hybrid studies [15], we performed GST pull-down assays, utilizing GST–Af9-(397–557) purified from *Escherichia coli* and *in vitro* translated Rnf2. GST empty vector was included as a negative control. As shown in Figure 1(B), Rnf2 bound the Af9 fusion protein, but not GST alone. The Rnf2 doublet likely reflects unmodified and autoubiquitinated forms of Rnf2 [23] generated during *in vitro* translation with the reticulocyte lysate system [24]. The results indicate that Rnf2 interacts with the C-terminus of Af9.

**Figure 2:**
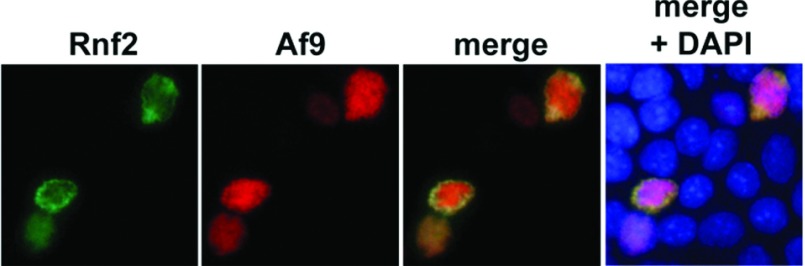
Rnf2 and Af9 colocalize in the nucleus of mIMCD3 cells mIMCD3 cells transiently co-transfected with constructs encoding pEGFP-Rnf2 and pDsRed-Af9 were counterstained with DAPI and examined by immunofluorescence microscopy at ×40. Representative images of three independent experiments are shown.

Interacting proteins with physiological relevance should colocalize within the cell. Accordingly, we performed colocalization experiments in mICMD3 cells. EGFP (enhanced green fluorescent protein)-Rnf2 was co-expressed with DspRed-tagged Af9 by transient transfection, and the expressed proteins were analysed by immunofluorescence microscopy. Nuclei were counterstained with DAPI. As shown in Figure 2, EGFP-Rnf2 and DspRed-tagged Af9 displayed nuclear co-localization. Whereas Af9 was homogeneously distributed in the nucleus, Rnf2 and the pool of co-localizing Af9 were apparent in a speckled distribution at the nuclear periphery (Figure 2). The speckled appearance of Rnf2 near the nuclear rim, contrasting with the more diffuse nuclear localization of Af9, suggests that Rnf2 and Af9 might interact in Polycomb bodies [25]. No cytoplasmic immunoreactivity for either protein was evident, in contrast to prior reports in HEK-293 (human embryonic kidney 293) T-cells, in which some cytoplasmic Af9 immunoreactivity was detected after transient transfection [22]. The neighbouring, unsuccessfully transfected cells, identified by DAPI (blue) staining of their nuclei, showed no fluorescence for either protein (Figure 2).

### Rnf2 represses basal and aldosterone-stimulated α-ENaC promoter activity and endogenous mRNA expression in mIMCD3 cells

**Figure 3:**
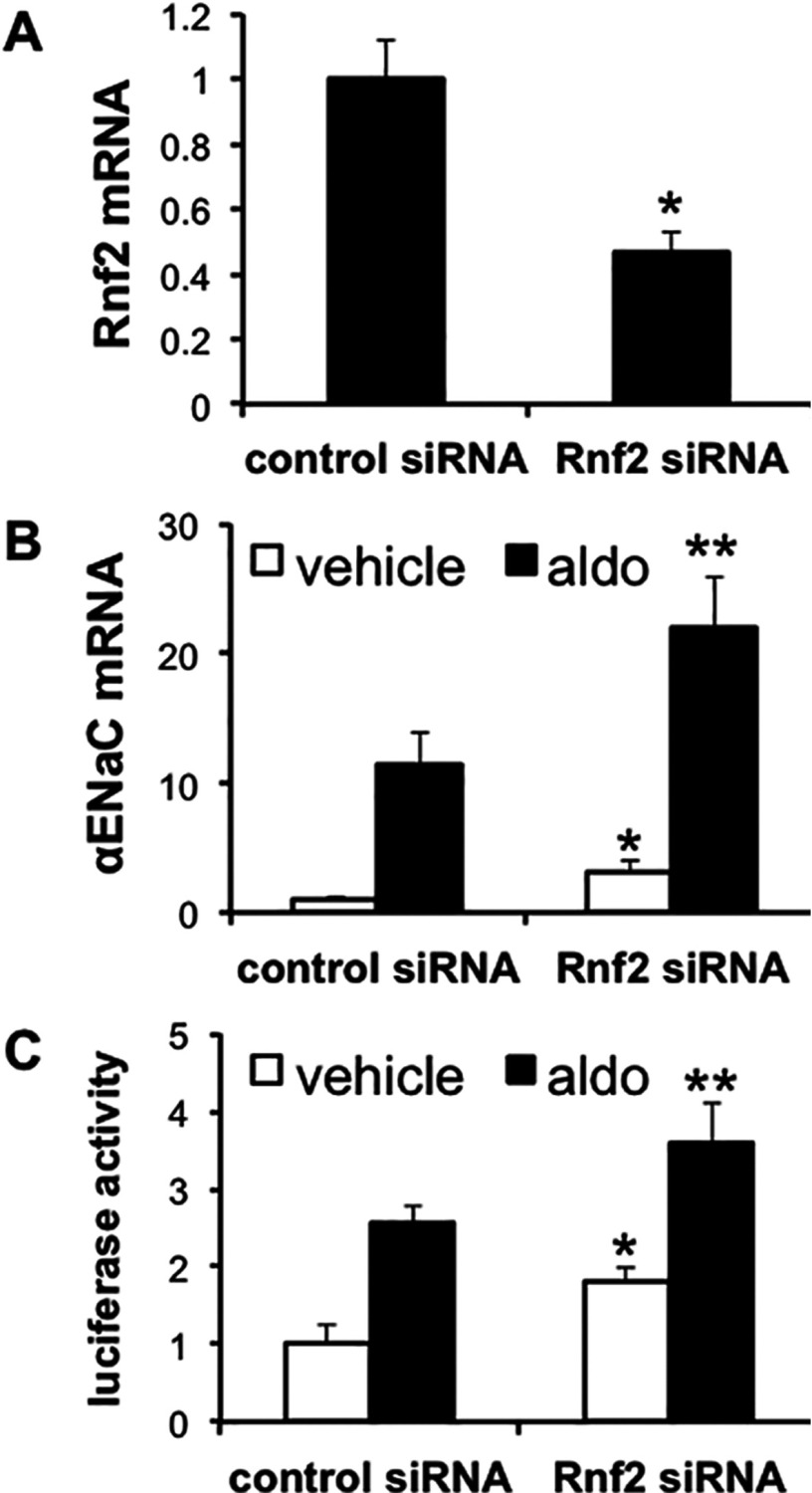
Rnf2 knock down enhances α-ENaC mRNA expression and promoter activity in mIMCD3 cells (**A**) mIMCD3 cells transfected with control or Rnf2-specific siRNAs were subjected to qRT-PCR analysis of Rnf2 and GAPDH mRNAs. Rnf2 mRNA was normalized to that of GAPDH. *P<0.05 compared with control, n=3. (**B**) mIMCD3 cells transfected as in (**A**) were treated with vehicle (white bars) or 1 μM aldosterone (aldo, black bars) for 24 h. α-ENaC and GAPDH mRNA levels were measured and normalized as in (**A**). *P<0.05 compared with vehicle, control siRNA; **P<0.05 compared with aldo, control siRNA, n=3. (**C**) mIMCD3 cells stably expressing an α-ENaC promoter-luciferase construct were transiently transfected and treated with vehicle (white bars) or aldo (black bars) as in (**B**). After 24 h, cell lysates were prepared for luciferase activity measurements, which were normalized to cell protein content. *P<0.05 compared with vehicle, control siRNA; **P<0.05 compared with aldo, control siRNA, n=3.

Given the robust expression of endogenous Rnf2 (Figure 1A), transient transfection of Rnf2 expression plasmids did not result in substantially enhanced and discriminating nuclear expression to test the functional consequences on basal α-ENaC transcription (results not shown, n=3). Instead, we exploited RNA interference to deplete the cells of Rnf2 and then measured the effects on endogenous α-ENaC mRNA expression, and, in separate experiments, on the activity of an α-ENaC promoter-luciferase construct stably incorporated in mIMCD3 cells under basal conditions or after treatment with aldosterone. qRT-PCR (quantitative real-time PCR) determinations indicated that Rnf2 mRNA expression in Rnf2 siRNA-transfected cells was approximately 55% less than in cells transfected with the control siRNA (Figure 3A). Consistent with prior studies [4], aldosterone treatment resulted in an about 11-fold increase in endogenous α-ENaC mRNA levels in the cells transfected with the control siRNA (Figure 3B). Cells transfected with Rnf2-specific siRNAs exhibited basal and aldosterone-stimulated α-ENaC mRNA levels that were approximately 3-fold and 2-fold greater, respectively, than controls (Figure 3B), indicating that native Rnf2 serves to repress basal, and to a lesser extent, aldosterone-stimulated α-ENaC mRNA expression. Similarly, transfection of the stable cell lines expressing α-ENaC promoter-luciferase with Rnf2 siRNA resulted in basal and aldosterone-stimulated promoter activities that were about 100% and about 50% greater, respectively, than similarly treated cells transfected with control siRNA (Figure 3C). Collectively, these results indicate that Rnf2 serves as a repressor of basal and, to a lesser extent, aldosterone-induced α-ENaC transcription in mIMCD3 cells.

### Rnf2, Af9 and the H2AK119ub1 and H3K27me3 chromatin marks are basally enriched at multiple subregions of the α-ENaC promoter

**Figure 4:**
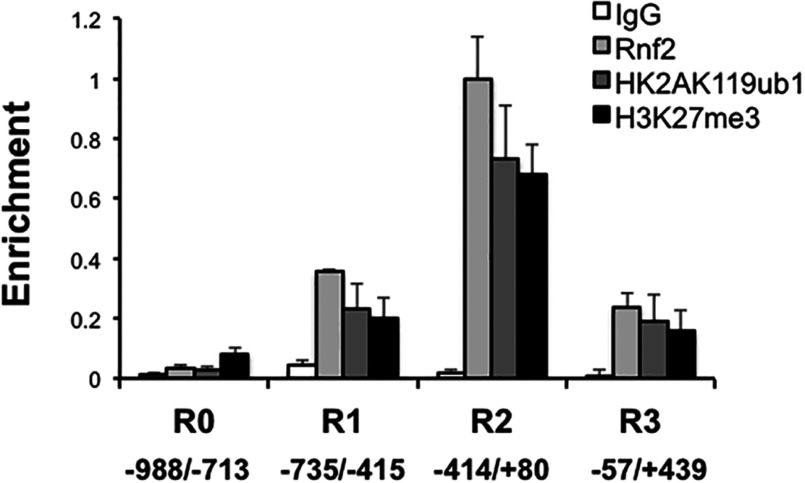
ChIP/QPCR analysis showing basal Rnf2 occupancy and H2AK119ub1 and H3K27me3 marks along the α-ENaC promoter Chromatin from mIMCD3 cells was IP with antibodies against Rnf2, H2AK119ub1, H3K27me3 or IgG. The ChIPs were quantified by QPCR with primers targeting the R0 (−988/−713), R1 (−735/−415), R2 (−414/+80), and R3 (−57/+439) subregions of the α-ENaC 5′-flanking region (n=3). The value for Rnf2-IP amplicon/input DNA obtained from the R2 subregion, which showed highest enrichment of all samples, was set to 1, and the other values were normalized to it. The values shown are the mean of triplicate determinations of three independent experiments. Error bars indicate±S.E.M.

Given the evidence for direct Rnf2–Af9 interaction (Figure 1B) and nuclear colocalization (Figure 2), the functional effects of Rnf2 knockdown on basal α-ENaC transcription (Figure 3C), and our prior demonstration that Af9 binds under basal conditions to each of the four partially overlapping subregions (R0, −988/−713; R1, −735/−415; R2, −414/+80; and R3, −57/+494) spanning the α-ENaC 5′-flanking region in mIMCD3 cells [6], we hypothesized that Rnf2 would also associate with one or more subregions of the α-ENaC promoter, and that these subregions would also bear H2AK119ub1 and H3K27me3 chromatin marks as indicators of Rnf2 and PRC2 activity, respectively. Accordingly, we used ChIP/QPCR to map the basal Rnf2 occupancy and H2AK119ub1 and H3K27me3 marks along the α-ENaC 5′-flanking region in mIMCD3 cells. As shown in Figure 4, Rnf2, H2AK119ub1 and H3K27me3 were associated to varying degrees with the R1–R3 subregions, and their relative abundance paralleled one another. The R0 subregion exhibited limited enrichment of Rnf2 and the two chromatin marks. The R1 and R3 subregions exhibited relatively comparable, higher levels of Rnf2, H2AK119ub1, and H3K27me3 enrichment. The R2 subregion exhibited even higher (~3-fold) levels of Rnf2, H2AK119ub1 and H3K27me3 enrichment.

### Rnf2 and Af9 co-occupy the α-ENaC promoter

**Figure 5:**
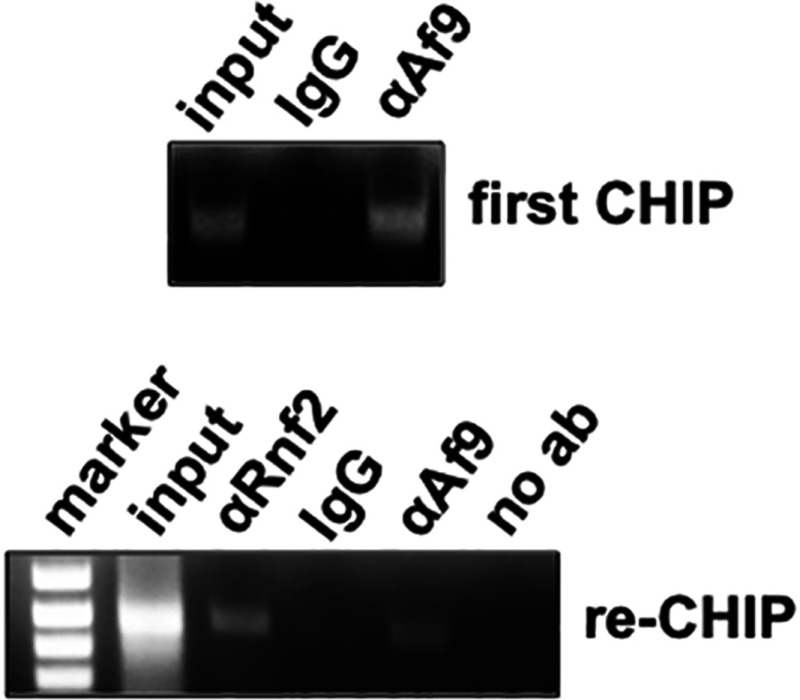
Sequential ChIP analysis showing co-occupancy of Rnf2 and Af9 at the R2 (−414/+80) subregion of the α-ENaC promoter Chromatin from mIMCD3 cells was sequentially IP with the Af9 antibody or IgG (‘First ChIP’), followed by re-ChIP with anti-Rnf2 antibody, Af9 (as a positive control) or IgG (as a negative control). Precipitated R2 subregion segments were amplified by PCR and resolved on agarose gels (representative of three independent experiments).

Given the physical interaction between Rnf2 and Af9 identified in the co-immunoprecipitation and GST pull-down assays, we sought evidence that the two proteins co-occupy the α-ENaC promoter in the context of chromatin in intact mIMCD3 cells. We focused our efforts on the R2 subregion, since it was the α-ENaC promoter subregion exhibiting the highest enrichment of Rnf2 under basal conditions (Figure 4). We performed ChIP/re-ChIP assays with antibodies to Af9 or Rnf2 (or IgG as a negative control) and α-ENaC primers targeting the R3 subregion of the α-ENaC promoter. The ChIPs with the Af9 antibody were re-immunoprecipitated with antibodies specific for Rnf2 or IgG. Agarose gel analysis of the ChIP and re-ChIP samples produced with the antibodies yielded the expected PCR products, whereas the IgG samples exhibited minimal signal (Figure 5). We conclude that Af9 and Rnf2 interact in chromatin associated with the R2 subregion of the α-ENaC promoter.

### Aldosterone inhibits Rnf2 occupancy, H2AK119 mono-ubiquitination and HK27 trimethylation at the α-ENaC promoter

**Figure 6:**
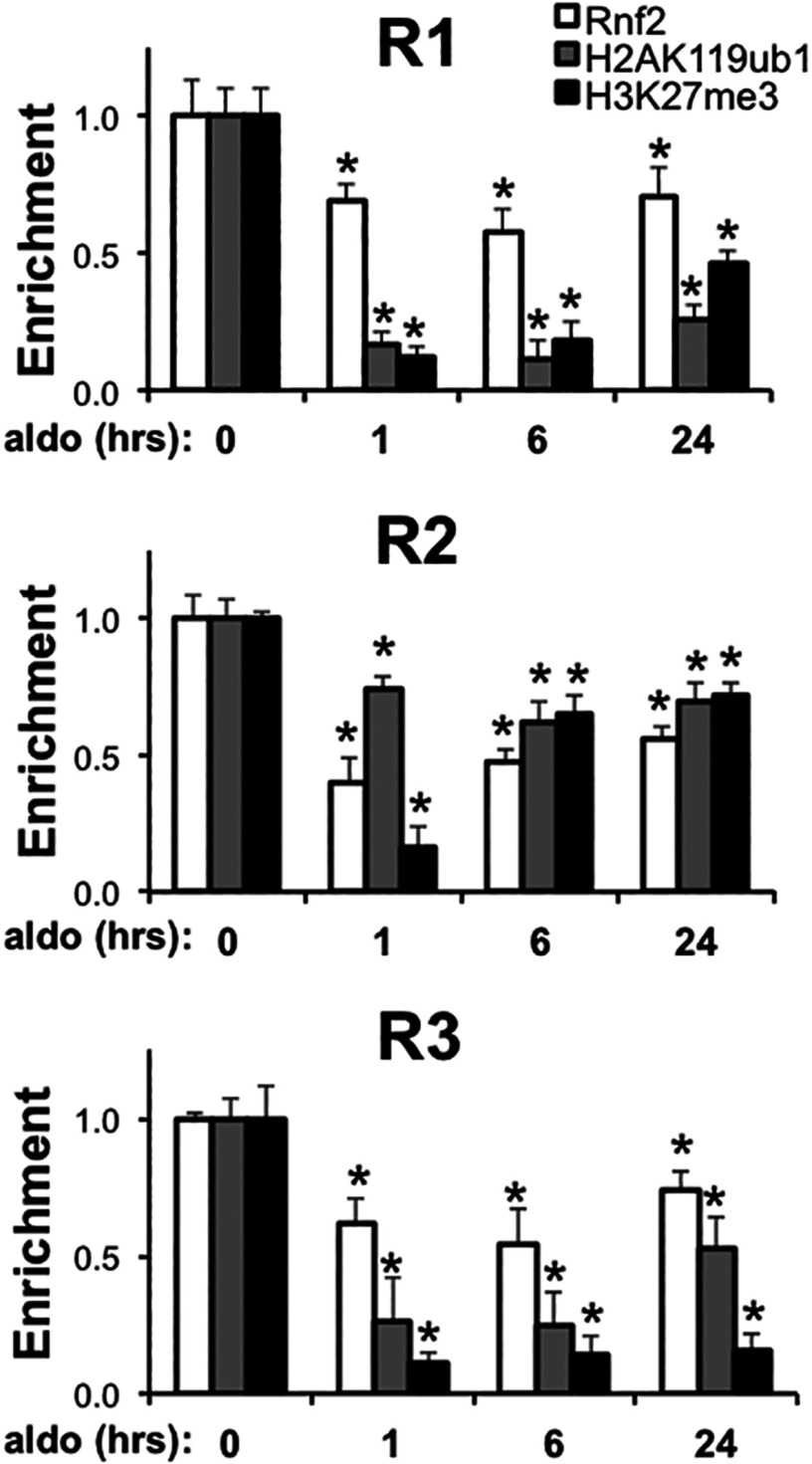
Aldosterone promotes early and sustained depletion of Rnf2, H2AK119ub1 and H3K27me3 from chromatin associated with the α-ENaC promoter mIMCD3 cells cultured in DMEM/F12 plus 10% charcoal-stripped serum for 16 h were treated with vehicle or 1 μM aldosterone (aldo) for the indicated time periods. ChIP/QPCR assays with antibodies specific for Rnf2, H2Ak119ub1 and H3K27me3 and primers to amplify the R1, R2 and R3 subregions of α-ENaC were performed. The aldo-treated samples were normalized to the vehicle time controls, which did not significantly vary over 24 h, and presented as the enrichment of the indicated proteins relative to values at time 0. Error bars indicate±S.E.M., n=3. *P<0.05 compared with time 0.

Given the interaction of Rnf2 with Af9 (Figure 1), the effects of Rnf2 knockdown to enhance basal and aldosterone-stimulated α-ENaC transcription (Figure 3), and the association of Rnf2 with the repressive chromatin marks H2AK119ub1 and H3K27me3 at the α-ENaC promoter under basal conditions (Figure 4), we hypothesized that aldosterone could trigger dispersal of Rnf2 from the promoter as it does with Af9 [6,7] and reprogram the local histone code to effect transcriptional de-repression. Accordingly, we performed a kinetic ChIP/QPCR analysis of Rnf2, H2AK119ub1 and H3K27me3 enrichment at the α-ENaC R1–R3 subregions following vehicle or aldosterone treatment of mIMCD3 cells for 1, 6 or 24 h. The R0 subregion was omitted from this analysis, because the very low basal amounts of Rnf2, H2AK119ub1 and H3K27me3 enrichment at the R0 subregion precluded discrimination. As seen in Figure 6, aldosterone treatment resulted in a significant loss of Rnf2, H2AK119ub1 and H3K27me3 in chromatin associated with α-ENaC R1–R3 subregions by 1 h, and this effect was maintained throughout the period studied. The decrements in Rnf2 were most prominent at the R1 and R2 subregions, whereas the losses of H2AK119ub1 and H3K27me3 were greatest in the R1 and R3 subregions (Figure 6).

## DISCUSSION

Understanding how genes move through distinct repressed, primed and active chromatin states is central to understanding transcriptional control processes that regulate gene induction. While much emphasis has been placed on the mechanisms of *trans*-activators and co-activators, much less is known about removal of co-repressors and repressive chromatin marks–‘de-repression’–as transcriptional control mechanisms. In previous work, we established that Af9 binds to multiple sites of the α-ENaC promoter to target Dot1a-mediated H3K79 hypermethylation for repression of basal α-ENaC transcription [6–8]. In this report, we identify Rnf2 as another Af9 binding partner involved in constraining basal transcription and in the aldosterone-induced de-repression of α-ENaC transcription. Specifically, we have shown by multiple independent assays (GST pull-down assay, co-immunoprecipitation and co-localization analyses and ChIP/re-ChIP assays) that Rnf2 directly interacts with Af9 *in vitro* and *in vivo* in renal CD cells. Knock down of Rnf2 mRNA expression by RNA interference increased expression of endogenous α-ENaC mRNA and the activity of an α-ENaC promoter-luciferase construct in mIMCD3 cells, consistent with a role of Rnf2 in basal repression of α-ENaC transcription. In ChIP/QPCR assays, Rnf2 associated with four defined subregions of the α-ENaC 5′-flanking region, previously shown to be enriched in Af9 [6–8], and was associated with enrichment of H3K27me3 and H2AK119ub1 associated with these sites *in vivo*. Kinetic studies demonstrated that aldosterone prompted loss of Rnf2 and the H3K27me3 and H2AK199ub1 chromatin marks beginning at 1 h, preceding the time course we previously described for loss of Af9, Dot1a, H3K79me3 (histone H3 lysine 79 trimethylated) and enrichment in RNA pol II [8]. We conclude that (1) Rnf2 physically and functionally interacts with Af9 in mIMCD3 cells; (2) PRC2-catalysed H3K27me3, together with Rnf2-mediated H2AK119ub1, are associated with basal repression of α-ENaC transcription in mIMCD3 cells; and (3) aldosterone depletes Rnf2 and reprograms these repressive histone marks to de-repress α-ENaC transcription.

Af9 was originally identified from human leukaemia samples containing a t(9,11)(p22;q23) translocation [26]. In addition to Dot1a, Af9 has been shown to interact in various cell types and assays with the planar cell polarity protein Diversin [27], specific isoforms of BCL-6 corepressor [15] and CDX8 [15], and to participate in a SEC (super elongation complex) containing the ELL family members ELL1, ELL2 and ELL3, the MLL translocation partners AFF1 (AF4/FMR2 family member 1) and AFF4, and the pol II elongation factor P-TEFb (positive transcription elongation factor b) [28]. Af9 interaction with partnering proteins appears to be mediated by an intrinsically disordered domain that assumes different conformations depending on the binding partner, thereby limiting binding to a single partner at any time [29]. Although Af9 interacts with the scaffolding protein AFF4 to comprise part of the SEC, it also exists outside the SEC when bound to Dot1L, which was found to inhibit SEC function [28]. Indeed, Dot1L and AFF4 directly compete for binding to Af9 [28]. This precedent for segregation of Af9 multi-protein complexes leads to the question of whether Rnf2, Dot1a, and Af9 coexist and function in a common ternary complex or as separate Rnf2–Af9 and Dot1a-Af9 complexes at the α-ENaC promoter. Further investigation will be needed to test whether Af9, through separate interactions with Rnf2 and with Dot1a, recruits these different chromatin modifiers to the α-ENaC promoter to constrain basal transcription.

Since Rnf2 and the PRCs lack inherent DNA-binding activity, other factors must be involved in targeted recruitment to promoters. In *Drosophila*, DNA Polycomb response elements and related targeting factors have been characterized. In mammals, PRC recruitment to genes is less well defined. One widely held model suggests that PRC2 generates H3K27me3 residues that serve as a platform for subsequent binding of PRC1 [17]. However, several examples of PRC1/H2AK119ub1 targeting independent of H3K27me3 have been described [30,31]. For example, Runx1 and CBFβ contribute to direct PRC1 recruitment at some promoter sites [32], and the lineage-specific transcription factor GATA-1 physically and functionally interacts with PRC2 during erythroid terminal maturation [33]. In the present report, Rnf2 association occurred at subregions that were marked by H3K27me3, but in a complex with the DNA-binding protein Af9.

While our pull-down assays with GST–Af9-(397-557) and *in vitro* translated Rnf2 indicate a direct protein–protein interaction, we cannot exclude the possibility that Rnf2 also directly interacts with other PcG proteins. For example, yeast two-hybrid studies testing interactions of PRC1 components demonstrated that a CBX8–Gal4 activation domain chimera interacted with Gal4 binding domain chimeras expressing either the carboxy-terminus of Af9 or Rnf2. However, no interaction was observed when the carboxy-terminus of Af9 was expressed as Gal4 binding domain chimera was tested with a Rnf2–Gal4 activation domain chimera. Co-expression of full-length CBX8 in yeast together with the Af9 and Rnf2 fusion proteins, yielded robust reporter gene activity, leading the authors to conclude that Af9 binding and Rnf2 do not directly interact, but assemble into a ternary complex through their binding to separate domains of CBX8 [15]. The reasons for the discrepancy between our GST pull-down results and the yeast two-hybrid studies are unclear, but may reflect conformational changes in the proteins related to the fused domains in the yeast two-hybrid experiments, or differences in yeast and mammalian systems.

The ChIP/QPCR analysis of the time-dependent effects of aldosterone on the histone code at the α-ENaC promoter (Figure 6), taken together with our previous analysis of the kinetics of Af9, H3K79me3, RNA pol II occupancy at the R3 subregion of the α-ENaC promoter [8], suggest a hierarchy of aldosterone-induced epigenetic reprogramming. Aldosterone-triggered decrements in Rnf2, H2AK119ub1 and H3K27me3 at the R3 subregion are apparent at 1 h of aldosterone exposure (Figure 6), whereas decrements in Af9 and H3K79me3 occur together with enhanced RNA pol II enrichment at 2 h. This suggests the possibility that aldosterone prompts a more rapid disruption of the Rnf2–Af9 interaction than of the Dot1a–Af9 interaction, as well as an inhibition of Ezh1/EZH2 activity and/or induction of demethylases that contribute to the overall level of H3K27me3 enrichment. Indeed, the steroid hormone progesterone was shown to induce total and phosphorylated EZH2 in mice [34].

The present study, combined with our earlier work on Dot1a and H3K79 methylation [4–8], expands the role of aldosterone as a versatile steroid hormone capable of modifying multiple effector proteins and histone marks of the epigenetic landscape. The aldosterone-stimulated epigenetic reprogramming across the subregions of the α-ENaC promoter to de-repress transcription–namely loss of Dot1a, Rnf2 and the H3K79me3, H2AK119ub1 and H327me3 marks–occurs in the context of roughly concurrent *trans*-activation mediated through the liganded MR binding to a GRE at -811 and Per1 binding to an E-box at −689 of the α-ENaC promoter [13,35]. Elucidating how these epigenetic de-repression and *trans-*activation events are choreographed should provide new insights into how other inducible genes are regulated.

## References

[1] Bubien J. K. (2010). Epithelial Na+ channel (ENaC), hormones, and hypertension. J. Biol. Chem..

[2] Christensen B. M., Perrier R., Wang Q., Zuber A. M., Maillard M., Mordasini D., Malsure S., Ronzaud C., Stehle J. C., Rossier B. C., Hummler E. (2010). Sodium and potassium balance depends on alpha-ENaC expression in connecting tubule. J. Am. Soc. Nephrol..

[3] Mick V. E., Itani O. A., Loftus R. W., Husted R. F., Schmidt T. J., Thomas C. P. (2001). The alpha-subunit of the epithelial sodium channel is an aldosterone-induced transcript in mammalian collecting ducts, and this transcriptional response is mediated via distinct cis-elements in the 5′-flanking region of the gene. Mol. Endocrinol..

[4] Zhang D., Li S., Cruz P., Kone B. C. (2009). Sirtuin 1 functionally and physically interacts with disruptor of telomeric silencing-1 to regulate alpha-ENaC transcription in collecting duct. J. Biol. Chem..

[5] Zhang W., Xia X., Jalal D. I., Kuncewicz T., Xu W., Lesage G. D., Kone B. C. (2006). Aldosterone-sensitive repression of ENaCalpha transcription by a histone H3 lysine-79 methyltransferase. Am. J. Physiol. Cell Physiol..

[6] Zhang W., Xia X., Reisenauer M. R., Hemenway C. S., Kone B. C. (2006). Dot1a-AF9 complex mediates histone H3 Lys-79 hypermethylation and repression of ENaCalpha in an aldosterone-sensitive manner. J. Biol. Chem..

[7] Zhang W., Xia X., Reisenauer M. R., Rieg T., Lang F., Kuhl D., Vallon V., Kone B. C. (2007). Aldosterone-induced Sgk1 relieves Dot1a-Af9-mediated transcriptional repression of epithelial Na +channel alpha. J. Clin. Invest..

[8] Zhang W., Yu Z., Wu H., Chen L., Kong Q., Kone B. C. (2013). An Af9 cis-element directly targets Dot1a to mediate transcriptional repression of the alpha-ENaC gene. Am. J. Physiol. Renal Physiol..

[9] Duarte J. D., Zineh I., Burkley B., Gong Y., Langaee T. Y., Turner S. T., Chapman A. B., Boerwinkle E., Gums J. G., Cooper-Dehoff R. M. (2012). Effects of genetic variation in H3K79 methylation regulatory genes on clinical blood pressure and blood pressure response to hydrochlorothiazide. J. Transl. Med..

[10] Kohler S., Pradervand S., Verdumo C., Merillat A. M., Bens M., Vandewalle A., Beermann F., Hummler E. (2001). Analysis of the mouse Scnn1a promoter in cortical collecting duct cells and in transgenic mice. Biochim. Biophys. Acta..

[11] Gumz M. L., Cheng K. Y., Lynch I. J., Stow L. R., Greenlee M. M., Cain B. D., Wingo C. S. (2010). Regulation of alpha-ENaC expression by the circadian clock protein Period 1 in mpkCCD(c14) cells. Biochim. Biophys. Acta..

[12] Gumz M. L., Stow L. R., Lynch I. J., Greenlee M. M., Rudin A., Cain B. D., Weaver D. R., Wingo C. S. (2009). The circadian clock protein Period 1 regulates expression of the renal epithelial sodium channel in mice. J. Clin. Invest..

[13] Stow L. R., Richards J., Cheng K. Y., Lynch I. J., Jeffers L. A., Greenlee M. M., Cain B. D., Wingo C. S., Gumz M. L. (2012). The circadian protein period 1 contributes to blood pressure control and coordinately regulates renal sodium transport genes. Hypertension.

[14] Ronzaud C., Loffing J., Bleich M., Gretz N., Grone H. J., Schutz G., Berger S. (2007). Impairment of sodium balance in mice deficient in renal principal cell mineralocorticoid receptor. J. Am. Soc. Nephrol..

[15] Hemenway C. S., de Erkenez A. C., Gould G. C. (2001). The polycomb protein MPc3 interacts with AF9, an MLL fusion partner in t(9;11)(p22;q23) acute leukemias. Oncogene.

[16] Margueron R., Reinberg D. (2011). The Polycomb complex PRC2 and its mark in life. Nature.

[17] Simon J. A., Kingston R. E. (2013). Occupying chromatin: Polycomb mechanisms for getting to genomic targets, stopping transcriptional traffic, and staying put. Mol. Cell.

[18] de Napoles M., Mermoud J. E., Wakao R., Tang Y. A., Endoh M., Appanah R., Nesterova T. B., Silva J., Otte A. P., Vidal M. (2004). Polycomb group proteins Ring1A/B link ubiquitylation of histone H2A to heritable gene silencing and X inactivation. Dev. Cell.

[19] Eskeland R., Leeb M., Grimes G. R., Kress C., Boyle S., Sproul D., Gilbert N., Fan Y., Skoultchi A. I., Wutz A., Bickmore W. A. (2010). Ring1B compacts chromatin structure and represses gene expression independent of histone ubiquitination. Mol. Cell.

[20] Stock J. K., Giadrossi S., Casanova M., Brookes E., Vidal M., Koseki H., Brockdorff N., Fisher A. G., Pombo A. (2007). Ring1-mediated ubiquitination of H2A restrains poised RNA polymerase II at bivalent genes in mouse ES cells. Nat. Cell Biol..

[21] Cao R., Wang L., Wang H., Xia L., Erdjument-Bromage H., Tempst P., Jones R. S., Zhang Y. (2002). Role of histone H3 lysine 27 methylation in Polycomb-group silencing. Science.

[22] Reisenauer M. R., Wang S. W., Xia Y., Zhang W. (2010). Dot1a contains three nuclear localization signals and regulates the epithelial Na+ channel (ENaC) at multiple levels. Am. J. Physiol. Renal Physiol..

[23] Buchwald G., van der Stoop P., Weichenrieder O., Perrakis A., van Lohuizen M., Sixma T. K. (2006). Structure and E3-ligase activity of the Ring–Ring complex of polycomb proteins Bmi1 and Ring1b. EMBO J..

[24] Iwamuro S., Saeki M., Kato S. (1999). Multi-ubiquitination of a nascent membrane protein produced in a rabbit reticulocyte lysate. J. Biochem..

[25] Pirrotta V., Li H. B. (2012). A view of nuclear Polycomb bodies. Curr. Opin. Genet. Dev..

[26] Iida S., Seto M., Yamamoto K., Komatsu H., Tojo A., Asano S., Kamada N., Ariyoshi Y., Takahashi T., Ueda R. (1993). MLLT3 gene on 9p22 involved in t(9;11) leukemia encodes a serine/proline rich protein homologous to MLLT1 on 19p13. Oncogene.

[27] Haribaskar R., Putz M., Schupp B., Skouloudaki K., Bietenbeck A., Walz G., Schafer T. (2009). The planar cell polarity (PCP) protein Diversin translocates to the nucleus to interact with the transcription factor AF9. Biochem. Biophys. Res. Commun..

[28] Luo Z., Lin C., Shilatifard A. (2012). The super elongation complex (SEC) family in transcriptional control. Nat. Rev. Mol. Cell Biol..

[29] Leach B. I., Kuntimaddi A., Schmidt C. R., Cierpicki T., Johnson S. A., Bushweller J. H. (2013). Leukemia fusion target AF9 is an intrinsically disordered transcriptional regulator that recruits multiple partners via coupled folding and binding. Structure.

[30] Dietrich N., Lerdrup M., Landt E., Agrawal-Singh S., Bak M., Tommerup N., Rappsilber J., Sodersten E., Hansen K. (2012). REST-mediated recruitment of polycomb repressor complexes in mammalian cells. PLoS Gen..

[31] Cao Q., Mani R. S., Ateeq B., Dhanasekaran S. M., Asangani I. A., Prensner J. R., Kim J. H., Brenner J. C., Jing X., Cao X. (2011). Coordinated regulation of polycomb group complexes through microRNAs in cancer. Cancer Cell.

[32] Yu M., Mazor T., Huang H., Huang H. T., Kathrein K. L., Woo A. J., Chouinard C. R., Labadorf A., Akie T. E., Moran T. B. (2012). Direct recruitment of polycomb repressive complex 1 to chromatin by core binding transcription factors. Mol. Cell.

[33] Ross J., Mavoungou L., Bresnick E. H., Milot E. (2012). GATA-1 utilizes Ikaros and polycomb repressive complex 2 to suppress Hes1 and to promote erythropoiesis. Mol. Cell Biol..

[34] Pal B., Bouras T., Shi W., Vaillant F., Sheridan J. M., Fu N., Breslin K., Jiang K., Ritchie M. E., Young M. (2013). Global changes in the mammary epigenome are induced by hormonal cues and coordinated by Ezh2. Cell Rep..

[35] Richards J., Greenlee M. M., Jeffers L. A., Cheng K. Y., Guo L., Eaton D. C., Gumz M. L. (2012). Inhibition of alpha-ENaC expression and ENaC activity following blockade of the circadian clock-regulatory kinases CK1delta/{varepsilon}. Am. J. Physiol. Renal Physiol..

